# Effect of Berberine Hydrochloride on the Diversity of Intestinal Flora in Parkinson's Disease Patients

**DOI:** 10.1155/2022/8381870

**Published:** 2022-05-30

**Authors:** Jiaojiao Li, Pin Meng, Jianyu Zhang, Mingli He

**Affiliations:** The First People's Hospital of Lianyungang, The First Affiliated Hospital of KangDA College of Nanjing Medical University, Lianyungang 222000, Jiangsu Province, China

## Abstract

This study aimed to investigate the effect of berberine hydrochloride on the diversity of intestinal flora in patients with Parkinson's disease (PD). Prospectively selected 68 PD patients, admitted to our hospital from April 2021 to June 2021, were randomly assigned to an observation group and a control group (*n* = 34 per group). Patients in the control group were given conventional treatment in accordance with Parkinson's diagnosis and treatment guidelines. Patients in the observation group were administered berberine hydrochloride besides the treatment in the control group. After continuous treatment for 3 months, the enzyme-linked immunosorbent assay was applied to determine interleukin-8 (IL-8), interleukin-6 (IL-6), and tumor necrosis factor-*α* (TNF-*α*) levels. High-throughput sequencing technology was employed to perform DNA sequencing on the 16S rRNA genes of all bacteria in stool samples before and after treatment in the two groups to analyze the distribution of intestinal flora. After treatment, the levels of IL-8, IL-6, and TNF-*α* were lower in the observation group than those in the control group (*P* < 0.05). Regarding intestinal flora, the Chao index, Ace index, and Shannon index were higher in the observation group than those in the control group. The Simpson index was lower in the observation group than that of the control group (*P* < 0.05). The principal component analysis chart analysis exhibited that the overall structure of the intestinal flora was quite different between the observation and the control groups after treatment. Berberine hydrochloride can improve the disorder of intestinal flora in PD patients and suppress the expression of inflammatory factors.

## 1. Introduction

Parkinson's disease (PD) is the second most common neurodegenerative disease affecting approximately 6.1 million people worldwide in 2021. PD is mainly manifested by the formation of Lewy bodies composed of the progressive loss of dopaminergic neurons in the midbrain substantia nigra and abnormal *α*-synuclein (*α*-Syn) aggregation [1]. The two-way communication exists between the central nervous system and the enteric nervous and autonomic nervous systems of the gastrointestinal tract [2], so there is gradually a deep understanding of the pathogenesis of PD. A previous study [3] has found that the abnormal folding and aggregation of *α*-Syn may originate from the enteric nervous system. Fayyad et al. [4] transplanted the gut microbes of healthy people and PD patients into sterile mice, and mice transplanted with the intestinal flora of PD patients presented dyskinesias and *α*-Syn synuclein aggregation. With the development of DNA sequencing, numerous clinical studies concern changes in the intestinal flora of PD patients. Saji et al. [5] reported that the abundance of Prevotaceae in the intestinal tract of PD patients was 77.6% lower than that of the healthy controls. The sensitivity of diagnosing PD could reach 86.1% when the relative abundance was 6.5% or even lower. Correlation analysis has shown that intestinal flora is associated with the patient's age and the severity of the disease. These studies have confirmed that the imbalance of the intestinal flora may be the initiating and continuing factors for the occurrence and development of PD, which provides a new direction for the treatment of PD.

Berberine hydrochloride, an effective ingredient extracted from the traditional Chinese medicine Rhizoma Coptidis, can be utilized to treat intestinal infections by adjusting and improving the intestinal microbial ecology. In AD, the effective ingredient of berberine hydrochloride berberine has been found to inhibit *β*/*γ*-secretion, increase *α*-secretion, and lessen A*β* levels in the hippocampus of AD mice, thereby improving cognitive impairment [6]. In patients with inflammatory bowel disease, berberine has been shown to stimulate dopamine *α*2 and *α*1 receptors [7]. The latest study [8] has confirmed that the striatal dopamine levels were elevated as measured by laser desorption mass spectrometry and berberine enhanced the imaging intensity of brain dopamine in mice receiving oral enterococci. These findings provide a theoretical basis for berberine to treat PD. Therefore, the present study analyzed the effect of berberine hydrochloride on the diversity of intestinal flora to further clarify the application value of berberine hydrochloride in PD patients.

## 2. Materials and Methods

### 2.1. Clinical Data

Inclusion criteria were as follows: meeting the diagnostic criteria for PD in the Guideline for the Treatment of Parkinson's Disease in China (Second Edition) [9], main manifestations include bradykinesia, resting tremor (4–6 Hz), and/or myotonia; distinct mind; and patients and their family members signed written informed consent. Exclusion criteria were as follows: Parkinsonism-plus syndrome, secondary Parkinsonism; combined with gastrointestinal disease; and allergic to the study drug. Sixty-eight PD patients admitted to our hospital between April 2021 and June 2021 were recruited in this study. The patients were randomly divided into observation and control groups (*n* = 34). No statistically significant difference in baseline data was detected between the two groups (*P* > 0.05), as given in Table 1.

### 2.2. Methods

The control group was given conventional treatment; in accordance with the Chinese Guideline for the Diagnosis and Treatment of Dementia and Cognitive Impairment in 2018 [10], to improve symptoms, work ability, and quality of life, anticholinergic drugs, amantadine, and dopamine receptor agonists were administered according to the patient's condition. In the observation group, besides the conventional treatment of the control group, berberine hydrochloride tablets (Sichuan Pacific Pharmaceutical Co., Ltd.; H51021485; 0.1 g/tablet) were administered orally, 0.2 g/time, 3 times/day. Patients of both groups were treated for 3 consecutive months.

### 2.3. Observation Indicators and Evaluation Indicators

1. Inflammation indicators: the patient's fasting cubital venous blood was collected in the morning before treatment and after 3 months of treatment, and the supernatant was taken after centrifugation. The levels of interleukin-8 (IL-8), interleukin-6 (IL-6), and tumor necrosis factor-*α* (TNF-*α*) were measured by ELISA using an automatic biochemical analyzer (model: Hitachi 7600).
2. Diversity of intestinal flora: stool specimens were collected before and 3 months after treatment. Intestinal flora detection was as follows: extraction of stool DNA, PCR amplification and purification, library construction, and sequencing. The detection of intestinal flora in the specimens was assisted by CNKINGBIO, Beijing, China.
3. Bacterial alpha-diversity analysis: Ace, Chao (evaluate the abundance of the flora), Shannon, and Simpson (evaluate the diversity of the flora) indices were calculated based on OTU cluster analysis results; the larger the index, the more abundant the species in the sample was.
4. Bacterial community structure *β*-diversity analysis: RDP Classifier software was utilized to compare the representative sequence of O-TUs with the Silva database, select species information with a reliability of more than 80%, and compare all sequences in each OTU. Based on OTU species identification, the flora species of the community structure were analyzed at the genus level.

### 2.4. Statistical Analysis

SPSS 21.0 software was employed for data analysis. Measurement data were represented by the mean ± SD. The difference between groups was compared by the *t*-test. Count data were represented by ((*n*) %) and analyzed using the *χ*^2^ test. Mothur software was applied to detect sample species abundance through MetaStat analysis (Shannon, Simpson, Ace, and Chao indices in alpha-diversity). *R* language was utilized to perform principal component analysis on intestinal flora *β*-diversity. A value of *P* < 0.05 was considered statistically significant.

## 3. Results

### 3.1. Comparison of the Levels of Inflammatory Factors between the Observation and Control Groups

The levels of IL-8, IL-6, and TNF-*α* were lower in the observation group than those in the control group after treatment (*P* < 0.05), as given in Table 2.

### 3.2. Comparison of Alpha-Diversity of Intestinal Flora between the Observation and Control Groups

**Figure 1:**
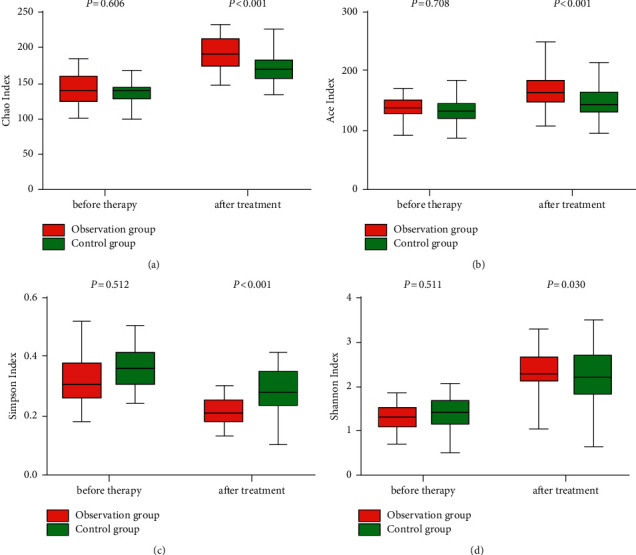
Comparison of alpha-diversity of intestinal flora between the observation and control groups.

Chao index, Ace index, and Shannon index were higher in the observation group than those of the control group, and Simpson index was lower in the observation group than that of the control group after treatment (*P* < 0.05), as given in Table 3 and Figure 1.

### 3.3. Beta-Diversity of Intestinal Flora in the Observation and Control Groups

**Figure 2:**
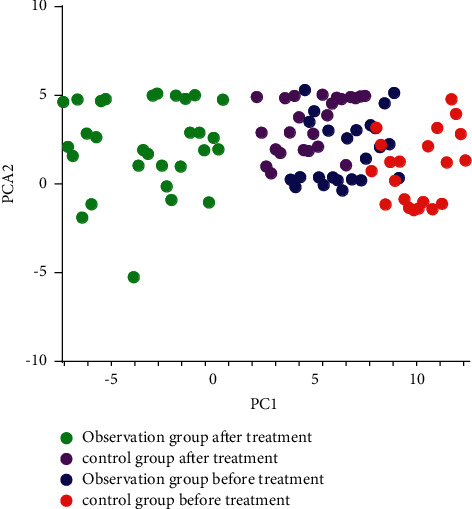
Analysis of *β*-diversity of intestinal flora.

Principal component analysis results exhibited that no significant difference in the overall structure of the intestinal flora was detected between the two groups before treatment. After treatment, the overall structure of the intestinal flora was quite different between the observation and the control groups, and the bacterial species were remarkably separated, as shown in Figure 2.

## 4. Discussion

PD is a degenerative disease of the nervous system. Substantia nigra dopaminergic neuron degeneration and striatal dopamine transmitter reduction are the main pathological features of PD. Early drug treatment can minimize the irreversible brain tissue and improve the prognosis. Clinically, anticholinergics, amantadine, and dopamine receptor agonists are usually administered according to the patient's condition to control motor and nonmotor symptoms. Clinical practice [11] has found that most PD patients have gastrointestinal dysfunction such as constipation and delayed gastric emptying, which occurs before the onset of motor symptoms. Wallen et al. [12] confirmed obvious intestinal flora imbalance in PD patients. These disorders alter the level of neuroactive substances and metabolites produced by the intestinal flora to a certain extent. Furthermore, the International Parkinson and Movement Disorder Society [13] positions the positive likelihood ratio of constipation to the diagnosis of prodromal PD at 2.2. Regarding this correlation, regulating the intestinal flora of PD patients has become a current research direction. Traditional Chinese medicine exerts an increasingly important effect on the treatment of PD. Berberine hydrochloride, also known as berberine, is a quaternary ammonium alkaloid isolated from Chinese medicines Rhizoma Coptidis and Cortex Phellodendri. Berberine hydrochloride has been widely concerned because it can effectively improve the structure of the intestinal flora and protect the damaged mucosal barrier of chronic diarrhea patients. Berberine hydrochloride has a beneficial effect on brain function [14]. Oral berberine hydrochloride can increase blood/fecal levodopa through intestinal bacteria in hyperlipidemia patients [15]. Berberine hydrochloride may improve brain function by upregulating the biosynthesis of levodopa in the intestinal flora through vitamin effects. Oral administration of berberine hydrochloride can trigger the biosynthesis of BH4 in the intestinal flora, increase the blood and brain dopa/dopamine concentration to enhance TH activity to produce L-dopa, and ultimately improve the animal's body function [8]. These results provide new ideas for controlling the crosstalk between the intestine and the brain and also provide a certain theoretical basis for the treatment of PD with berberine hydrochloride.

Phenylalanine hydroxylase catalysis and tyrosine-dopa-dopamine conversion are completed in the brain. The interruption of this pathway is an important reason for the occurrence and development of PD [16] and can cause intestinal flora disorders. The homeostasis of the intestinal biota is destroyed, and numerous pathogenic bacteria can cause intestinal mucosal dysfunction, increase damage to the brain, and lead to a vicious circle of disease [17]. Sequencing of different variable regions of most PD patients who use bacterial 16s ribosomal RNA has demonstrated that the intestinal flora is remarkably different from that of normal peers [18]. The decrease of *Bacillus faecalis* in the intestinal mucosa and the increase of *Ralstonia* of PD patients promote the change of the microbial balance in the colon to an inflammation-prone phenotype; this kind of microbial imbalance can cause incomplete intestinal mucosa and intestinal barrier dysfunction, which can directly increase the level of bacterial metabolites (such as endotoxin) in blood, trigger an inflammatory response in the central nervous system, and promote pathological *α*-Syn accumulation, which can further activate glial cells, trigger the continuous inflammatory response of the central nervous system, and ultimately promote the occurrence and development of PD. Therefore, it is important to understand the diversity of the intestinal flora of such patients and to suppress this feedforward response. It is found that the treatment of berberine hydrochloride not only improves the body's insulin resistance but also dramatically reduces the relative abundance of bacteria that produced branched-chain amino acids in a diabetic patient [19]. A previous study [20] has shown that berberine can regulate intestinal flora by promoting the catabolism of branched-chain amino acids in the liver and adipose tissue. In this study, the recovery of the flora diversity of patients treated with berberine hydrochloride was better, as shown in Figures 1 and 2. This may be because berberine hydrochloride provides H through dihydroberberine and promotes the production of large amounts of BH4 by dihydrobiopterin. The increase in BH4 can enhance TH activity and accelerate L-dopa production by intestinal bacteria to improve intestinal flora.

Growth evidence supports the involvement of neuroinflammatory response in the pathogenesis of PD [21]. TNF, as a proinflammatory factor and immunomodulator with multiple biological activities, can mediate mucosal damage. Especially, TNF-*α* can activate epithelial cells and promote the accumulation of neutrophils in the colonic mucosa. Moreover, basic research [22] has shown that TNF-*α* can promote the production of inflammatory mediators IL-8 and IL-6, make the body's anti-inflammatory/proinflammatory state imbalance, and aggravate intestinal mucosal damage. Poudel et al. [23] confirmed that berberine hydrochloride has a beneficial effect on amyloid cells and the disrupted metabolic processes in activated microglia. Berberine hydrochloride can decrease the neuroinflammatory response by suppressing the activation of the NF-*κ*B signaling pathway in AD patients [24]. This study has also demonstrated that berberine hydrochloride can remarkably improve the inflammation level of PD patients. This may be because berberine hydrochloride is the isoquinoline alkaloid extracted from Chinese medicine Rhizoma Coptidis, and its active ingredients can decrease the intestinal mucosal damage caused by nonsteroidal anti-inflammatory drugs and chronic stress and reduce the expression of inflammatory mediators in the body.

## 5. Conclusion

In summary, berberine hydrochloride can suppress the expression of inflammatory factors in PD patients and improve the disorder of intestinal flora. Since the PD intestinal flora may promote the occurrence of misfolding of *α*-synuclein, the study concerning the PD intestinal flora and inflammatory cytokines may further supplement the therapeutic mechanism of berberine hydrochloride. Nevertheless, it has not been discussed in this study due to the limited sample size. On this point, the sample size needs to be further expanded for the in-depth study to provide a theoretical basis for the treatment of PD.

## Figures and Tables

**Table 1:** Comparison of baseline data between the observation and control groups.

Group	Sex (male/female)	Mean age ($\bar{x}$ ± *s*, year)	Mean duration ($\bar{x}$ ± *s*, year)	Parkinson's disease evaluation scale score ($\bar{x}$ ± *s*, score)	Family history (*n*, %)
Observation (*n* = 34)	20/14	52.67 ± 7.68	5.58 ± 1.51	35.26 ± 7.26	7 (20.59)
Control (*n* = 34)	22/12	53.53 ± 8.29	5.77 ± 1.82	36.98 ± 8.03	9 (26.47)
*t/x* ^*2*^	0.249	0.444	0.468	0.926	0.327
*P*	0.318	0.659	0.641	0.358	0.567

**Table 2:** Comparison of the levels of inflammatory factors between the observation and control groups ($\bar{x}$ ±*s*, *μ*g/L).

Group	IL-8		IL-6		TNF-*α*	
	Before treatment	After treatment	Before treatment	After treatment	Before treatment	After treatment
Observation (*n* = 34)	62.26 ± 10.26	42.35 ± 10.24^*∗*^	58.26 ± 13.65	33.98 ± 11.21^*∗*^	25.62 ± 6.98	14.36 ± 4.29^*∗*^
Control (*n* = 34)	60.29 ± 11.57	47.88 ± 10.25^*∗*^	59.32 ± 14.27	40.26 ± 12.20^*∗*^	26.54 ± 7.01	16.23 ± 4.54^*∗*^
*t*	0.743	2.226	0.313	2.210	0.542	2.307
*P*	0.460	0.030	0.755	0.031	0.589	0.023

^*∗*^ *P* < 0.05 vs. the same group before treatment.

**Table 3:** Comparison of the alpha-diversity of the intestinal flora between the observation and control groups ($\bar{x}$ ±*s*).

Group	Chao index		Ace index		Simpson index		Shannon index	
	Before treatment	After treatment	Before treatment	After treatment	Before treatment	After treatment	Before treatment	After treatment
Observation (*n* = 34)	141.22 ± 20.36	193.65 ± 26.31^*∗*^	136.23 ± 21.34	167.19 ± 23.42^*∗*^	0.33 ± 0.09	0.22 ± 0.05^*∗*^	1.36 ± 0.33	2.29 ± 0.46^*∗*^
Control (*n* = 34)	139.34 ± 22.98	169.27 ± 21.98^*∗*^	134.87 ± 19.68	152.26 ± 24.67^*∗*^	0.34 ± 0.08	0.28 ± 0.06^*∗*^	1.40 ± 0.41	2.11 ± 0.51^*∗*^
*t*	0.518	5.566	0.376	3.641	0.660	6.604	0.660	2.204
*P*	0.606	<0.001	0.708	<0.001	0.512	<0.001	0.511	0.030

^*∗*^ *P* < 0.05 vs. the same group before treatment.

## Data Availability

The data used to support the findings of this study are available from the corresponding author upon request.
